# General Commentary: Immune-metabolic crosstalk in HNSCC: mechanisms and therapeutic opportunities

**DOI:** 10.3389/fonc.2025.1662713

**Published:** 2025-09-12

**Authors:** Jiayi Chen

**Affiliations:** Department of Stomatology, Suzhou Wujiang District Hospital of Traditonal Chinese Medicine, Suzhou, China

**Keywords:** HNSCC, TME, ICB, metabolic reprogramming, crosstalk

Dear Editor, Professor Lee Peng Karen-Ng,

I read with great interest the recently published review by Qin et al. titled *“Immune-metabolic crosstalk in HNSCC: mechanisms and therapeutic opportunities”* in *Frontiers in Oncology* (1). I would like to commend the authors for their comprehensive and insightful synthesis of current knowledge on the complex interplay between metabolic reprogramming and immune regulation in HNSCC (Head and neck squamous cell carcinoma).

This review effectively highlighted the metabolic adaptations within the tumor microenvironment—such as glycolysis, fatty acid oxidation, amino acid metabolism, and adenosine signaling—and their roles in shaping immunosuppressive landscapes. The authors also underscored the relevance of key signaling pathways (PI3K/Akt/mTOR, NF-κB, PD-1/PD-L1) that linked metabolic and immune modulation, offering a compelling rationale for combinatorial therapeutic strategies.

Importantly, the review identified several potential therapeutic targets and ongoing clinical trials, reflecting the translational potential of immunometabolic interventions in HNSCC. This focused on actionable targets, such as glycolysis inhibitors, adenosine pathway antagonists, and mTOR inhibitors, was particularly timely given the challenges of resistance to current ICB (Immune checkpoint blockade) therapies.

While the review was thorough, I would like to offer a few additional suggestions:

Though the introduction of ICB therapies (e.g., anti PD-1/PD-L1 agent) to oncology can transform the management of various malignancies, including HNSCC, the application of anti PD-1/PD-L1 agent alone cannot achieve favorable survival rate of patients with targeted cancer (2). As authors say, amino acid plays a vital role in immune regulation in HNSCC. Arginase-1 is a cytosolic enzyme originally isolated from the liver and found in the TME of most solid tumors, including HNSCC (3). It not only has direct effects on tumor growth, but also further fosters tumorigenesis by inducing immune suppression (4). OATD-02, a new boronic acid derivative, is the only dual inhibitor, which can address the benefits of pharmacological inhibition of arginase 1 and 2 in cancer (5). Whether OATD-02 can enhance the anti-cancer effect of ICB therapies on HNSCC through immune-metabolic crosstalk signal should considered in the future. First, three group OATD-02+ICB, ICB, and control will be set for cell apoptosis, proliferation, and drug resistance trials in HNSCC cells. Next, co-cultivation of T-cell and tumor cell can be conducted to examine the bioactivity of immune cells. In addition, PI3K-AKT-mTOR signaling should be implemented to test for activation. Finally, bare mouse tumor formation test can be conducted *in vivo*.

Overall, authors have produced a highly valuable reference for clinicians and researchers aiming to develop new strategies to overcome immunotherapy resistance in HNSCC.
