# Correction: General Commentary: Immune-metabolic crosstalk in HNSCC: mechanisms and therapeutic opportunities

**DOI:** 10.3389/fonc.2026.1806068

**Published:** 2026-02-17

**Authors:** Jiayi Chen

**Affiliations:** Department of Stomatology, Suzhou Wujiang District Hospital of Traditional Chinese Medicine, Suzhou, Jiangsu, China

**Keywords:** NHANES, neurology, logistic (logit) regression, cross-sectional study, cognitive function

The title of this article was erroneously given as: “General Commentary: The letter to editor Immune-metabolic crosstalk in HNSCC: mechanisms and therapeutic opportunities”. The correct title of the article is “General Commentary: Immune-metabolic crosstalk in HNSCC: mechanisms and therapeutic opportunities”.

The original version of this article has been updated.

